# Are two really better than one? A retrospective study comparing monotherapy versus combination therapy for *Stenotrophomonas maltophilia* infections

**DOI:** 10.1128/spectrum.03475-25

**Published:** 2026-02-24

**Authors:** Natalie Harris, Amy Mackowiak, Rebekah H. Wrenn, Hui-Jie Lee, Alexander Reed, Alaattin Erkanli, Nicholas A. Turner, Rebekah Moehring, Connor R. Deri

**Affiliations:** 1 Department of Pharmacy, Duke University Hospital 22957, Durham, North Carolina, USA; 2 Division of Infectious Diseases, Duke University School of Medicine 12277 , Durham, North Carolina, USA; 3 Biostatistics & Bioinformatics, Duke University School of Medicine 189407, Durham, North Carolina, USA; Univ of Texas Southwestern Medical Center, Dallas, Texas, USA

**Keywords:** resistance, *Stenotrophomonas maltophilia*, combination therapy, monotherapy

## Abstract

Management of *Stenotrophomonas maltophilia* infections is challenging. The Infectious Diseases Society of America recommends up-front combination therapy, though this may increase toxicity without clear benefit. We compared combination therapy (CT) versus monotherapy (MT) in the management of *S. maltophilia* infections to inform institutional decision-making and microbiology reporting. We conducted a retrospective study of adults with *S. maltophilia* infections who received ≥72 h of organism-directed, *in vitro* active therapy between July 1, 2019, and November 30, 2024. Patients were excluded if they lacked evidence of infection, had cystic fibrosis, or had polymicrobial cultures. CT was defined as more than one agent given concomitantly for ≥72 h. The primary outcome was clinical failure, a composite of 30-day all-cause mortality and microbiologically confirmed recurrence. Of 370 patients screened, 103 met the inclusion criteria (86 MT and 17 CT). CT recipients were more often immunocompromised (70.6% vs 22.1%) with bacteremia (58.8% vs 19.8%). Clinical failure occurred in 47.1% versus 36.0% of patients in the CT and MT cohorts, respectively (RD: 15.6%; 95% CI: −9.9% to 41.2%). Resistance developed in 8.7% of isolates, most commonly with levofloxacin. Adverse effects occurred in 26.7% MT and 35.3% CT cohorts. We observed no differences in clinical failure between CT versus MT for the management of *S. maltophilia* infections. Risks of adverse effects with CT should be balanced with perceived benefit based on host factors, infection source, and risk of resistance. These findings suggest MT is a viable option for select patients with *S. maltophilia* infections.

**IMPORTANCE:**

Management of *Stenotrophomonas maltophilia* infections is challenging due to intrinsic antimicrobial resistance. Current Infectious Diseases Society of America guidance recommends up-front combination therapy for all cases based on limited observational and *in vitro* data. In our retrospective cohort study comparing combination therapy with monotherapy, we found no significant differences in 30-day clinical failure but observed a numerically higher rate of adverse events with combination therapy. These results fill an important gap in real-world outcomes and support monotherapy as a viable and potentially safer option. Our findings may guide clinical decision-making and microbiology reporting toward more individualized treatment strategies.

## INTRODUCTION

*Stenotrophomonas maltophilia* is an aerobic, glucose non-fermenting, gram-negative bacillus that may cause colonization or infection, particularly in vulnerable hosts with underlying lung disease or hematologic malignancies (1). *S. maltophilia* produces biofilm and virulence factors that, when acting as a true pathogen, can cause significant morbidity and mortality in immunocompromised hosts (1). In addition to the diagnostic challenge of determining infection versus colonization, *S. maltophilia* exhibits resistance factors that preclude the use of common antimicrobials (2). Treatment selection is hampered by multiple resistance mechanisms, including genes and gene mutations that render most conventional β-lactams ineffective, intrinsic resistance to aminoglycosides, and accumulation of multidrug efflux pumps that reduce activity of trimethoprim-sulfamethoxazole (TMP/SMX), tetracyclines, and fluoroquinolones (2).

Historically, disease severity and clinical progression guided the recommended antibiotic treatment for *S. maltophilia* infections. Prior to 2023, the Infectious Diseases Society of America (IDSA) guidance recommended the use of monotherapy (MT) for mild infections and urged consideration of combination therapy (CT) in moderate to severe infections unresolved with one agent alone (3). However, in 2023, the Clinical Laboratory and Standards Institute (CLSI), which provides guidance on antimicrobial susceptibility testing and interpretation, recommended caution with the use of both levofloxacin and TMP/SMX monotherapy given evidence of treatment-emergent resistance and a lack of pharmacodynamic data for these agents, respectively (4–6). Despite hesitation with levofloxacin, TMP/SMX monotherapy has remained the mainstay of treatment for *S. maltophilia* infections due to its high *in vitro* susceptibility, extensive clinical experience, exceptional epithelial lining fluid (ELF) concentrations, and a dual mechanism of action with synergistic potential, which contributes to its ability to evade resistance mechanisms (3, 7, 8).

In 2023, the IDSA updated its “Guidance on the treatment of antimicrobial resistant gram-negative infections,” suggesting upfront combination therapy for all *S. maltophilia* infections until clinical improvement due to the absence of data supporting any individual agent (9). In 2024, IDSA guidance provided a hierarchy of appropriate agents for combination therapy: (i) cefiderocol, (ii) minocycline, (iii) TMP/SMX, and (iv) levofloxacin (8). They list the combination of ceftazidime-avibactam and aztreonam as a second preferred option for management of *S. maltophilia* infections (10). This recommendation was made based on limited observational and *in vitro* data. Potential harms of exposure to dual agents may include higher rates of toxicity and resistance, without clear additional clinical benefit (11, 12). Thus, whether combination therapy should be approached as a sequential add-on strategy versus an empiric treatment strategy for patients with *S. maltophilia* infections remains an open discussion among infectious diseases experts.

TMP/SMX has been considered the first-line agent for *S. maltophilia* within the Duke University Health System and retains 99% susceptibility in aggregate data. The aim of this study was to assess institutional data on combination therapy (CT) versus monotherapy (MT) antimicrobial regimens in the management of *S. maltophilia* infections to guide decision-making on microbiology lab reporting and first-line therapy recommendations.

## MATERIALS AND METHODS

### Design, setting, and participants

We conducted a multi-site, retrospective, observational cohort study of adult patients ≥18 years old with *S. maltophilia* infection at Duke University Hospital, Duke Raleigh Hospital, or Duke Regional Hospital between July 1, 2019, and November 30, 2024. Patients were included if they received at least 72 hours of organism-directed, *in vitro*, active therapy defined as: TMP/SMX, levofloxacin, minocycline, cefiderocol, or aztreonam in combination with ceftazidime/avibactam (CAZ/AVI). Given breakpoint changes that occurred during the study period, patients receiving ceftazidime therapy were included only if concomitantly receiving therapy with an *in vitro*, active agent listed above. Patients were excluded if they did not exhibit clinical signs or symptoms of infection, if cultures were polymicrobial, if they were pregnant or breastfeeding, or if they had a history of cystic fibrosis. Polymicrobial infections were defined as the growth of a clinically relevant organism in the index culture where *S. maltophilia* was isolated. Due to the subjectivity of identifying patients with true infection, we conducted a robust process of exclusion. If the diagnosis was unclear for the primary reviewer after robust chart review, two pharmacists would review independently to determine if the isolate was likely colonization versus true infection. In cases where the two independent pharmacist reviewers disagreed, cases were reviewed with two physicians until a consensus was reached among the group.

Patients were included in the CT cohort if they received >1 organism-directed, *in vitro* active agent for ≥72 h concomitantly. Patients receiving ceftazidime with an *in vitro* active agent were included in the MT cohort, unless they would otherwise meet criteria outlined above for inclusion in the CT cohort. In patients with more than one episode of *S. maltophilia* infection, only the first episode per hospitalization was analyzed in our study. The follow-up period was 30 days, and only data in existence as of December 31, 2024, were collected and used in the study.

### Measures

The primary endpoint was clinical failure defined as a composite of all-cause 30-day mortality and 30-day microbiologically confirmed recurrence, comparing the CT versus MT cohorts. Microbiologically confirmed recurrence was defined as infection recurrence with a positive culture from the same site as the index culture. Secondary endpoints included 30-day mortality, 30-day infection-related mortality, 30-day infection recurrence, and the rate of resistance to index treatment within 30 days. Infection recurrence was defined as the recurrence of clinically significant signs/symptoms related to the index infection with *S. maltophilia*. Other endpoints of interest included adverse events to antibiotic therapy and antibiotic regimens used. Adverse events included acute kidney injury (AKI), defined as an increase in Scr by ≥0.3 mg/dL or ≥1.5 times the baseline value; cytopenias, defined as a hemoglobin <7 g/dL or a platelet count <150,000/µL; QTc prolongation, defined as an increase of QTc to >500 ms or >60 ms from baseline; and hypersensitivity or gastrointestinal upset as documented during the index encounter. The following subgroup analyses were also conducted to compare CT versus MT of the primary endpoint: high (≥2) qPITT score, immunocompromised, age ≥65, bloodstream infection, respiratory infection, and skin and soft tissue infection.

### Statistical analysis

Categorical variables were summarized as frequency and percentage, and continuous variables were summarized as mean, standard deviation (SD), median, quartile 1 (Q1), quartile 3 (Q3), and range. The 30-day clinical failure rate was calculated as the proportion of patients with 30-day clinical failure. The 30-day clinical failure rates were compared between cohorts overall and within subgroups by unadjusted risk difference (RD) using the MT cohort as the reference, and its 95% confidence interval (CI) from normal approximation. Additionally, a multivariable linear Poisson model with robust standard errors was fit to estimate the adjusted RD in 30-day clinical failure rates between cohorts, adjusting for qPITT bacteremia score (13). We included qPITT (≥2 vs <2) in the regression model based on preliminary analyses that showed qPITT was the least balanced between MT and CT groups. All analyses were conducted in R Version 4.4.0.

## RESULTS

**Fig 1:**
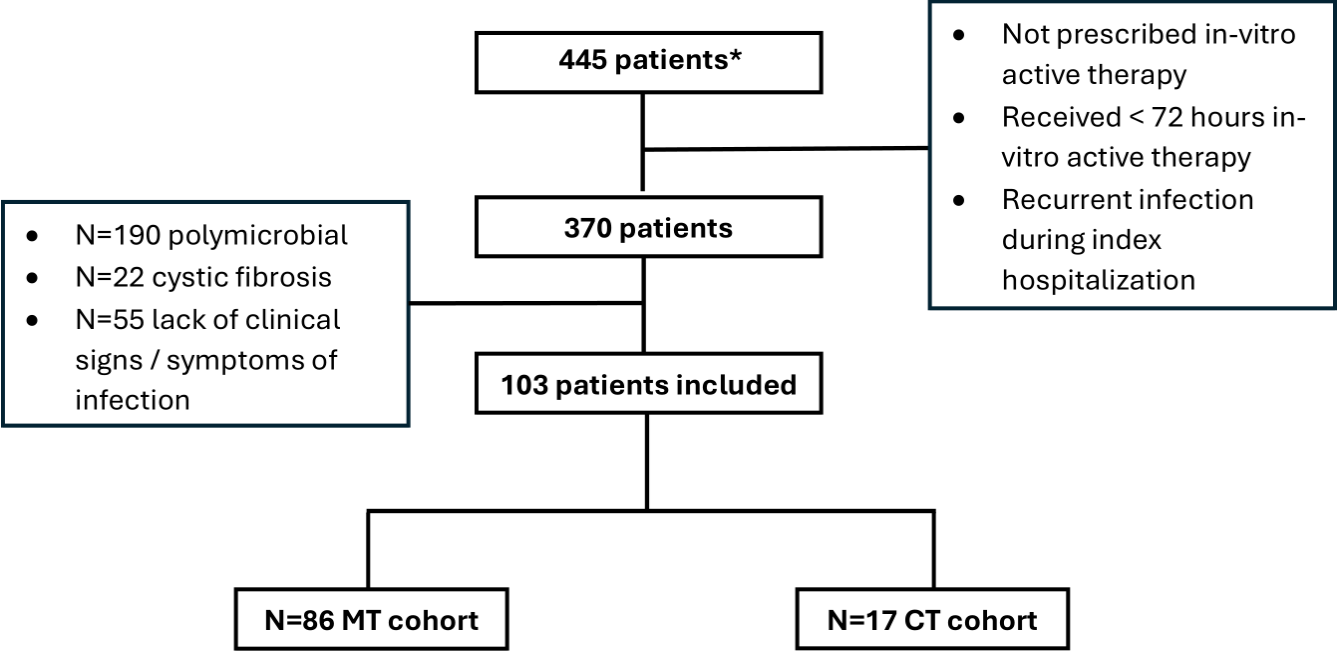
Selection of study population. *Initial 445 patients identified through an electronic health record query that captured patients with a culture positive for *S. maltophilia* during the study period. CT, combination therapy; MT, monotherapy.

Among 445 patients with *S. maltophilia* in cultures, a total of 103 patients met inclusion criteria with 86 (83.5%) in the MT group and 17 (16.5%) in the CT group. Most exclusions were due to polymicrobial culture data (Fig. 1). Baseline patient and infection characteristics are summarized in Table 1. The mean age of patients included was 57.3 years, and the majority of patients were seen at our academic medical center, Duke University Hospital (*n* = 91, 88.3%). At the time of culture collection, most patients had markers of severe illness: the mean qPITT bacteremia score was 1.5 (SD = 1.3), 65% (*n* = 67) of patients were admitted to the intensive care unit (ICU), and 47.6% (*n* = 49) required mechanical ventilation. Patients in the CT group were more likely to have an indwelling line (70.6% vs 58.1%) and be immunocompromised (70.6% vs 22.1%) compared to the MT group. Active malignancy was the most common reason for being immunocompromised (*n* = 15, 14.6%), followed by solid organ transplant (*n* = 13, 12.6%) and hematopoietic stem cell transplant (*n* = 2, 1.9%). Five patients in both the CT and MT cohorts had a history of lung transplant (29.4% vs 5.8%). The most common source of infection among both cohorts was respiratory (*n* = 60, 58.3%); however, patients in the CT group were more likely to have positive blood cultures (58.5% vs 19.8%) and central-line associated infection (29.4% vs 9.3%). Infectious disease specialists were consulted in 100% of the CT group compared with 68.6% (*n* = 59) of the MT group. At baseline, 100% of the tested isolates were susceptible to TMP/SMX (*n* tested = 103), minocycline (*n* tested = 19), and cefiderocol (*n* tested = 5), while 89.3% (*n* tested = 103) tested susceptible to levofloxacin. Susceptibility data were unavailable for CAZ/AVI and aztreonam. Thirty-six patients overall (*n* = 28, 32.6% in MT vs *n* = 8, 47.1% in CT) were receiving therapy for an unrelated, concomitant infection, with pneumonia (*n* = 12, 11.7%), and bloodstream infection (*n* = 10, 9.7%) being the most common indications.

**TABLE 1:** Baseline patient characteristics^*a*^

Characteristic	MT (*N* = 86)	CT (*N* = 17)	Total (*N* = 103)
Patient characteristics			
Age, years, mean (SD)	57.8 (14.1)	54.6 (16.1)	57.3 (14.4)
≥65 years, *n* (%)	33 (38.4)	5 (29.4)	38 (36.9)
<65 years, *n* (%)	53 (61.6)	12 (70.6)	65 (63.1)
Male, *n* (%)	48 (55.8)	11 (64.7)	59 (57.3)
Race, *n* (%)			
White	52 (60.5)	10 (58.8)	62 (60.2)
Black or African American	31 (36.0)	6 (35.3)	37 (35.9)
Asian	0 (0)	1 (5.9)	1 (1.0)
Unknown	3 (3.5)	0 (0)	3 (2.9)
Ethnicity, *n* (%)			
Not Hispanic or Latino	77 (89.5)	15 (88.2)	92 (89.3)
Hispanic or Latino	4 (4.7)	0 (0)	4 (3.9)
Unknown	5 (5.8)	2 (11.8)	7 (6.8)
Hospital, *n* (%)			
Duke University Hospital	74 (86.0)	17 (100)	91 (88.3)
Duke Raleigh Hospital	7 (8.1)	0 (0)	7 (6.8)
Duke Regional Hospital	5 (5.8)	0 (0)	5 (4.9)
qPITT bacteremia score, mean (SD)	1.6 (1.4)	1.2 (1.0)	1.5 (1.3)
qPITT ≥ 2, *n* (%)	41 (47.7)	6 (35.3)	47 (45.6)
qPITT < 2, *n* (%)	45 (52.3)	11 (64.7)	56 (54.4)
Charlson comorbidity index, mean (SD)	4.1 (2.4)	4.1 (2.1)	4.1 (2.4)
Hospital LOS, median (IQR)	20.2 (10.4–34.0)	38.1 (11.4–51.2)	21.4 (10.5–38.4)
ICU admission, *n* (%)	57 (66.3)	10 (58.8)	67 (65.0)
Mechanical ventilation, *n* (%)	44 (51.2)	5 (29.4)	49 (47.6)
Presence of an indwelling device, *n* (%)			
Indwelling central line	50 (58.1)	12 (70.6)	62 (60.2)
Cardiovascular device	16 (18.6)	2 (11.8)	18 (17.5)
Prosthetic valve	3 (3.5)	0 (0)	3 (2.9)
Prosthetic joint	5 (5.8)	0 (0)	5 (4.9)
Other	10 (11.6)	1 (5.9)	11 (10.7)
Renal dysfunction, *n* (%)	30 (34.9)	5 (29.4)	35 (34.0)
Chronic kidney disease	14 (16.3)	2 (11.8)	16 (15.5)
Hemodialysis	4 (4.7)	0 (0)	4 (3.9)
Acute kidney injury	15 (17.4)	3 (17.6)	18 (17.5)
Continuous renal replacement therapy	3 (3.5)	0 (0)	3 (2.9)
COPD, *n* (%)	17 (19.8)	1 (5.9)	18 (17.5)
Bronchiectasis or structural lung disease, *n* (%)	9 (10.5)	1 (5.9)	10 (9.7)
Transplant, *n* (%)	9 (10.5)	6 (35.3)	15 (14.6)
Hematopoietic stem cell transplant, *n* (%)	1 (1.2)	1 (5.9)	2 (1.9)
Allogeneic	1 (1.2)	1 (5.9)	2 (1.9)
Solid organ transplant, *n* (%)^*b*^	8 (9.3)	5 (29.4)	13 (12.6)
Heart	2 (2.3)	1 (5.9)	3 (2.9)
Lung	5 (5.8)	5 (29.4)	10 (9.7)
Liver	1 (1.2)	0 (0)	1 (1.0)
Active malignancy receiving chemotherapy, *n* (%)	9 (10.5)	6 (35.3)	15 (14.6)
Leukemia	0 (0)	2 (11.8)	2 (1.9)
Lymphoma	0 (0)	1 (5.9)	1 (1.0)
Other	9 (10.5)	3 (17.6)	12 (11.7)
Infection characteristics			
Source of infection, *n* (%)			
Respiratory	53 (61.6)	7 (41.2)	60 (58.3)
Skin and soft tissue	12 (14.0)	0 (0)	12 (11.7)
Central line associated	8 (9.3)	5 (29.4)	13 (12.6)
Intra-abdominal	3 (3.5)	4 (23.5)	7 (6.8)
Urinary	5 (5.8)	1 (5.9)	6 (5.8)
Source of culture growth, *n* (%)			
Respiratory—all	53 (61.6)	7 (41.2)	60 (58.3)
Respiratory—sputum	10 (11.6)	1 (5.9)	11 (10.7)
Respiratory—BAL	20 (23.3)	6 (35.3)	26 (25.2)
Respiratory—ETS	19 (22.1)	0 (0)	19 (18.4)
Blood	17 (19.8)	10 (58.8)	27 (26.2)
Tissue	15 (17.4)	1 (5.9)	16 (15.5)
Intra-abdominal	1 (1.2)	0 (0)	1 (1.0)
Urine	4 (4.7)	0 (0)	4 (3.9)
Infectious diseases consulted, *n* (%)	59 (68.6)	17 (100)	76 (73.8)

^*a*^ BAL, bronchoalveolar lavage; COPD, chronic obstructive pulmonary disease; CT, combination therapy; ETS, endotracheal swab; ICU, intensive care unit; LOS, length of stay; MT, monotherapy.

^*b*^ One patient included in the CT cohort was a heart-lung transplant.

**Fig 2:**
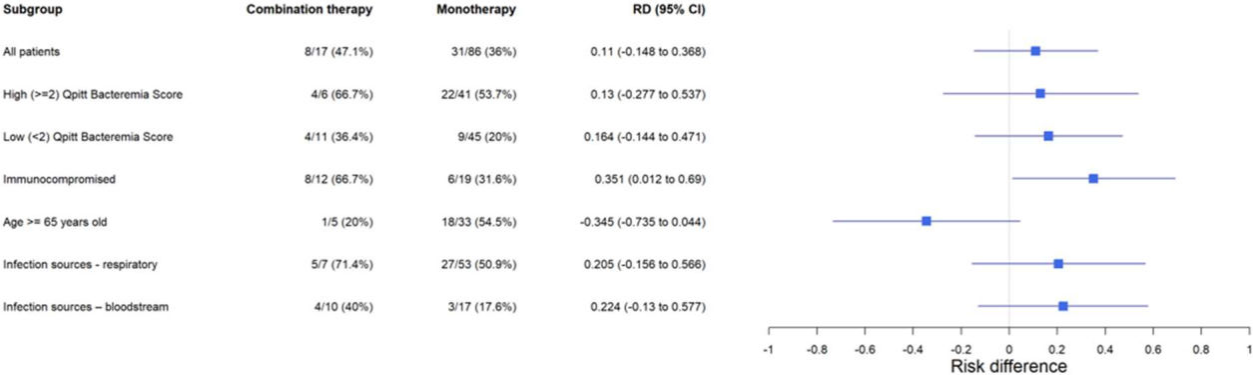
Unadjusted risk difference (RD) for primary composite outcome in prespecified subgroups. Presented as *n* (%) with unadjusted risk difference (RD) and 95% confidence intervals (CI). On the forest plot, the line of no effect is on 0; <0 favors combination therapy (CT), and >0 favors monotherapy (MT) regimens.

All primary and secondary outcomes are summarized below in Table 2. Thirty-one patients (36%) in the MT group versus 8 patients (47.1%) in the CT group met the primary composite outcome of clinical failure (unadjusted RD = 11%; 95% CI = [−15% to 37%]), defined as 30-day all-cause mortality and 30-day microbiologically confirmed recurrence. Within the components of the composite outcome, 30-day all-cause mortality occurred in 29.4% (*n* = 5) CT cohort versus 27.9% (*n* = 24) MT cohort, and mortality was deemed to be infection-related in 11.8% (*n* = 2) and 10.5% (*n* = 9), respectively. In a prespecified subgroup analysis of the primary composite outcome, 66.7% (*N* = 8) of the immunocompromised patients in the CT group versus 31.6% (*N* = 6) in the MT group experienced clinical failure (unadjusted RD = 35%; 95% CI = [1% to 69%]). The percentage of patients who experienced clinical failure and unadjusted RDs (95% CIs) for all prespecified subgroups are summarized in Fig. 2. After multivariable modeling including qPITT score, the adjusted risk difference for the primary composite of clinical failure was aRD 15% (95% CI = [−9.9% to 41.2%]).

**TABLE 2:** Outcomes^*a*^

Outcome	MT (*N* = 86)	CT (*N* = 17)	Unadjusted RD (95% CI)	Adjusted RD^*b*^ (95% CI)
30-day clinical failure, *n* (%)	31 (36.0)	8 (47.1)	11% (−15% to 37%)	15% (−9.9% to 41.2%)
All-cause mortality	24 (27.9)	5 (29.4)		
Microbiologic confirmed recurrence	10 (11.6)	3 (17.6)		
30-day infection related mortality, *n* (%)	9 (10.5)	2 (11.8)		
30-day infection recurrence, *n* (%)	30 (34.9)	10 (58.8)		
30-day microbiological recurrence, *n* (%)	12 (14.0)	3 (17.6)		
Resistance within 30 days, *n*/*N* (%)^*c*^	7/15 (46.7)	2/3 (66.7)		
Levofloxacin	4/7 (57.1)	2/3 (66.7)		
Minocycline	1/1 (100)	1/1 (100)		
TMP/SMX	0/10 (0)	0/2 (0)		
Adverse effects related to therapy, *n* (%)	23 (26.7)	6 (35.3)		
Cytopenia	2 (2.3)	0 (0)		
Leading to discontinuation, *n/N (%)*	2/2 (100)	0 (0)		
Gastrointestinal	4 (4.7)	2 (11.8)		
Leading to discontinuation, *n*/*N* (%)	2/4 (50)	0 (0)		
QTc prolongation	0 (0)	2 (11.8)		
Leading to discontinuation, *n*/*N* (%)	0 (0)	1/2 (50)		
Hypersensitivity	1 (1.2)	0 (0)		
Leading to discontinuation, *n*/*N* (%)	1/1 (100)	0 (0)		
Acute kidney injury	14 (16.3)	2 (11.8)		
Leading to discontinuation, *n*/*N* (%)	7/14 (50)	1/2 (50)		

^*a*^ CT, combination therapy; MT, monotherapy.

^*b*^ The adjusted risk difference of the primary outcome was completed through adjusting for qPITT < 2.

^*c*^ Resistance within 30 days calculated based on a denominator of isolates with available data on repeat cultures.

Two isolates (11.8%) in the CT cohort versus seven isolates (8.1%) in the MT cohort developed resistance to initial antibiotics within 30 days, with levofloxacin resistance being the most common overall. Adverse events occurred in 35.3% (*n* = 6) patients in the CT group versus 26.7% (*n* = 23) MT group and led to treatment discontinuation in 11.8% (*n* = 2) and 15.1% (*n* = 13), respectively. The most common documented adverse effects (ADEs) were acute kidney injury (*n* = 16, 15.5%), cytopenias (*n* = 2, 1.9%), and QTc prolongation (*n* = 2, 1.9%).

The most common antimicrobial used in the MT group was TMP/SMX (*n* = 42, 48.8%) followed by levofloxacin (*n* = 34, 39.5%). Common CT regimens included levofloxacin + minocycline (*n* = 7, 41.2%) and TMP/SMX + minocycline (*n* = 4, 23.5%). Antimicrobial regimens are described in Table 3. Antimicrobial agents used over time and the temporal relationship between CT versus MT use are described in Fig. S1 and S2 of the supplemental material, respectively.

**TABLE 3:** Antibiotic regimens used^*a*^

Antibiotic regimen	MT (*N* = 86)	CT (*N* = 17)
TMP/SMX, *n* (%)	42 (48.8)	
Levofloxacin, *n* (%)	34 (39.5)	
Minocycline, *n* (%)	6 (7.0)	
Ceftazidime/avibactam + aztreonam, *n* (%)	3 (3.5)	
Cefiderocol, *n* (%)	1 (1.2)	
Levofloxacin + minocycline, *n* (%)		7 (41.2)
TMP/SMX + minocycline, *n* (%)		4 (23.5)
Minocycline + cefiderocol, *n* (%)		2 (11.8)
TMP/SMX + levofloxacin, *n* (%)		1 (5.9)
TMP/SMX + levofloxacin + cefiderocol, *n* (%)		1 (5.9)
TMP/SMX + levofloxacin + minocycline, *n* (%)		1 (5.9)
TMP/SMX + levofloxacin + minocycline + cefiderocol, *n* (%)		1 (5.9)

^*a*^ CT, combination therapy; MT, monotherapy; TMP/SMX, trimethoprim/sulfamethoxazole.

## DISCUSSION

In our retrospective cohort study, we observed clinical failure rates that were numerically higher with CT compared to MT in the management of *S. maltophilia* infections, although this difference was not statistically significant. Additionally, CT was associated with a higher incidence of adverse effects (ADEs), including acute kidney injury which resulted in therapy discontinuation in 50% of affected patients. We saw minimal emergence of resistance to primary monotherapy options such as TMP/SMX in our study, which suggests MT remained effective and safe for most patients with *S. maltophilia* infections. There was an under-representation of patients on CT regimens from Duke Raleigh and Duke Regional community hospitals, likely attributable to low representation of patient populations more likely to receive CT within our institution (i.e., high severity of illness and immunocompromising condition). To our knowledge, this is one of the few available studies to directly compare CT versus MT for *S. maltophilia* infection from all infectious sources in a real-world cohort, incorporating clinical outcomes, safety data, and microbiological findings.

The overwhelming majority of data available to guide recommendations for up-front CT in *S. maltophilia* infections is *in vitro*. Emerging retrospective studies have found no significant differences in 7-day clinical response (12), all-cause 30-day mortality (14), clinical cure (15), or in-hospital mortality (12) with CT versus MT in *S. maltophilia* infections (12, 14, 15). However, Chen et al. observed a difference in 30-day mortality in the immunocompromised subgroup in favor of CT (15). In contrast, our study found higher rates of clinical failure in immunocompromised patients receiving CT, reflecting the greater severity of illness and tendency to prescribe more aggressive regimens in this population. Our findings align with growing evidence supporting MT as a viable and potentially safer management option in *S. maltophilia* infections. Almangour and colleagues observed increased rates of acute kidney injury (AKI) in patients receiving CT (9% vs 18%; odds ratio 0.35; 95% CI = [0.16–0.78]) (16). The rate of AKI in our cohort is likely attributable, in part, to the institutional use of TMP/SMX, which was administered to 48.5% of patients included. The severity and clinical consequences of ADEs in the management of an infection with limited therapeutic options are further illustrated by the high rate of therapy discontinuation following AKI among affected patients. Our study adds to the available literature evaluating CT versus MT for the management of *S. maltophilia* infections, but unlike most of the published data, which is restricted to respiratory sources, our study is inclusive of all infectious sources. Moreover, our findings offer insight into current prescribing patterns, including the characteristics of patients selected for CT and their associated outcomes, which may inform future investigations of treatment decisions and patient selection strategies.

Despite recent guidance from both IDSA and CLSI, we observed no emergent resistance to TMP/SMX in our cohort, and this remains an attractive MT option at our institution. The IDSA guidance panel urged against TMP/SMX monotherapy for *S. maltophilia* infections, citing pharmacokinetic studies that question its bactericidal activity despite favorable minimum inhibitory concentrations (MICs) (10). Wei et al. found no single agent exhibited bactericidal activity against 12 *S. maltophilia* isolates, while some CT regimens showed enhanced bacterial killing when at least one agent was active (17). However, the clinical relevance of these findings remains uncertain due to limited clinical trial data. In a retrospective cohort study by Watson et al., monotherapy with TMP/SMX was associated with higher mortality compared with fluoroquinolones (31.3% vs 13.6%) and prolonged hospitalization (18). This study is limited by the inability to distinguish colonization from true infection, incomplete antimicrobial susceptibility testing data, and inclusion of polymicrobial infections, all of which challenge the correlation of TMP/SMX in *S. maltophilia* infections with worse clinical outcomes (18). With nearly half of our MT cohort receiving TMP/SMX, we found similar clinical outcomes and no emergence of resistance, while excluding colonization and polymicrobial infections which might confound these outcomes. Other small observational studies have compared TMP/SMX monotherapy to alternative agents, including levofloxacin and minocycline, and demonstrate similar efficacy between groups (5, 19). In a more recent retrospective cohort study, there was no difference overall in rates of clinical cure for *S. maltophilia* pneumonia between MT with minocycline or TMP/SMX (OR = 0.92; 95% CI = 0.34–2.44); however, they did observe a higher incidence of in-hospital mortality in the TMP/SMX group (19). We note that this was performed at a single center, and there was no observed difference in infection-related mortality (*P* = 0.62), rates of clinical cure were lower in patients receiving <12 mg/kg/day TMP/SMX, and pneumonia recurrence was higher in the minocycline cohort (19). The limited available data for TMP/SMX MT are conflicting, and institution-specific review is vital for local guidance.

Given the limited comparative data on outcomes between individual MT regimens for *S. maltophilia,* our analysis provides observational evidence on how these agents performed in clinical practice. Further, our findings reinforced the continued role of TMP/SMX as a trusted therapeutic option in contrast to levofloxacin, where our observed rising resistance may limit utility. Nearly 11% of isolates tested resistant to levofloxacin at baseline, and of remaining isolates with baseline susceptibility and available data after completion of therapy, 60% developed resistance to levofloxacin. This finding supports the action of our microbiology laboratory to add a comment on all cultures with *S. maltophilia* urging against the use of levofloxacin MT, in alignment with CLSI recommendations. In consideration of a similar comment for TMP/SMX, our findings that TMP/SMX retains *in vitro* activity against *S. maltophilia* and patients on MT had similar outcomes make us hesitant to accept this suggested comment for all clinical scenarios. Based on our results, our institution will opt for a more nuanced approach of encouraging the use of empiric CT for immunocompromised patients or for patients with invasive *S. maltophilia* infections, as these patients are at highest risk of true invasive infection and aggressive therapy up front is warranted. Patient selection is a crucial factor in determining appropriate therapy for *S. maltophilia* infections, considering the potential for colonization versus true infection and ADEs of commonly used agents. Future studies could provide further insight into optimal antimicrobial dosing for *S. maltophilia*, patient or infection characteristics where CT is beneficial, and the nature of differentiating colonization from true infection with *S. maltophilia* growth in culture.

Our study has limitations. Due to the limited sample size, we did not have the statistical power to show MT is not inferior to CT through the non-inferiority hypothesis testing, nor could we adjust for more than one covariate in the regression analysis. Given the retrospective nature of our study, we were unable to account for variables that could influence the decision for MT versus CT during the index encounter; however, we excluded patients with documented or suspected *S. maltophilia* colonization. Additionally, group sizes and characteristics were unbalanced. A higher proportion of patients in the CT cohort were immunocompromised, had documented bacteremia, and catheter-related bloodstream infections. Lastly, the exclusion of polymicrobial infections and cystic fibrosis limits generalizability in clinical practice; however, in these settings, clinical outcomes are often driven by factors other than *S. maltophilia* isolation.

### Conclusion

In a cohort of 103 patients with *S. maltophilia* infections, we observed a numerically higher 30-day clinical failure rate in CT versus MT regimens, which was not statistically different. Our findings support the use of MT in select patients with *S. maltophilia* infections given retained susceptibility of first-line agents such as TMP/SMX and the additive risk of adverse effects with CT. We consider comorbidities, risk of adverse medication effects, immune status, and clinical severity of the patient when designing a therapeutic regimen for the management of *S. maltophilia* infections.

## SUPPLEMENTAL MATERIAL

Supplemental Material spectrum.03475-25-s0001.docx Fig. S1 and S2.

ASM does not own the copyrights to Supplemental Material that may be linked to, or accessed through, an article. The authors have granted ASM a non-exclusive, world-wide license to publish the Supplemental Material files. Please contact the corresponding author directly for reuse.
