# Supplementary material for: Are two really better than one? A retrospective study comparing monotherapy versus combination therapy for Stenotrophomonas maltophilia infections
Source: Microbiol Spectr. 2026 Feb 24;14(4):e03475-25. doi: 10.1128/spectrum.03475-25 (PMC13055297; doi:10.1128/spectrum.03475-25)
Supplement: Supplemental Material — Fig. S1 and S2. [file spectrum.03475-25-s0001.docx]

**Are Two Really Better than One? A Retrospective Study Comparing Monotherapy versus Combination Therapy for *Stenotrophomonas maltophilia* Infections**

Natalie Harris, PharmD^a^#*; Amy Mackowiak, PharmD, BCIDP^a^; Rebekah H. Wrenn, PharmD, BCIDP^a,b^; Hui-Jie Lee, Ph.D.^c^; Alexander Reed, MS^c^; Alaattin Erkanli, Ph.D.^c^; Nicholas A. Turner, MD, MHSc, FACP^b^; Rebekah Moehring, MD, MPH^c^; Connor R. Deri; PharmD, BCIDP^a^

1. Department of Pharmacy, Duke University Hospital, Durham, NC, USA
2. Division of Infectious Diseases, Duke University School of Medicine, Durham, NC, USA
3. Biostatistics & Bioinformatics, Duke University School of Medicine, Durham, NC, USA

Running Head: Monotherapy vs Combination therapy in *S. maltophilia*

#Address correspondence to Natalie Harris, nmharris22@outlook.com

*Present address: Natalie Harris, Prisma Health Richland Hospital, Columbia, SC, USA

**SUPPLEMENTARY MATERIAL FOR PUBLICATION**

**Figure S1: Antibiotic Selection for Definitive Therapy Over Time**

*Figure S1: Definitive therapy defined as antibiotic received to complete treatment course with all available microbiology data. Antibiotics were counted individually, and counts include both definitive monotherapy and combination therapy regimens.

Definitions: FDC: cefiderocol; MIN: minocycline; TMP/SMX: trimethoprim/sulfamethoxazole; LVX: levofloxacin; CZA: ceftazidime/avibactam; AZT: aztreonam; CZD: ceftazidime

**Figure S2: Use of Combination Therapy Over Time**

Figure S2 describes the number of patients receiving combination therapy versus the number of patients receiving monotherapy over time, compared to the total number of patients included in the cohort study. Note: 2024 data is incomplete, collected through 11/2024.
